# Household and Environmental Typhoid Fever Transmission in Bandar Lampung City, Indonesia: A Case-Control Study

**DOI:** 10.31729/jnma.8744

**Published:** 2024-09-30

**Authors:** Prayudhy Yushananta, Muflichah Febriani Eka Putri

**Affiliations:** 1 Department of Environmental Health, Tanjungkarang Health Polytechnic, Lampung, Indonesia

**Keywords:** *case-control study*, *food contamination*, *handwashing*, *risk factor*, *sanitation*, *typhoid fever*

## Abstract

**Introduction:**

Typhoid fever is an endemic disease that causes substantial morbidity and mortality in low and middle-income countries. A case-control study was conducted to understand the risk factors for typhoid fever.

**Methods:**

The study applied the case-control method to compare past exposure between participants who had typhoid fever (cases) and participants who did not have typhoid fever (controls) after obtaining ethical approval (No. 244/KEPK-TJK/III/2023). We identified and surveyed patients with typhoid fever confirmed by blood culture. Control subjects were randomly selected neighbors of cases with no history of typhoid fever. Both cases and controls were interviewed at home. During the visit, a structured observation of their living environment was performed. Multivariable analysis was applied using logistic regression and odds ratio to evaluate the relationship between exposure and risk of typhoid fever. Data collected was entered into Statistical Package for Social Science (SPSS) after being checked for completeness, edited, and coded.

**Results:**

The study found that typhoid fever was significantly associated with not washing hands with soap (Adjusted Odds Ratio = 6.08; 95% CI 2.43 - 15.21), eating unwashed raw vegetables (Adjusted Odds Ratio = 4.63; 95% CI 1.62 - 11.73); eating mobile food (Adjusted Odds Ratio = 10.89; 95% CI 4.08 - 29.05); eating street food (Adjusted Odds Ratio = 3.28; 95% CI 1.27 - 8.45); no access to safe drinking water (Adjusted Odds Ratio = 6.08; 95% CI 2.11 - 17.52); no access to healthy latrines (Adjusted Odds Ratio = 3.59; 95% CI 1.47 - 8.78).

**Conclusions:**

The study found that typhoid fever was associated with inadequate food and personal hygiene, and poor housing.

## INTRODUCTION

Typhoid fever remains a major cause of morbidity and mortality in many low- and middle-income countries.^1,2^ In Indonesia, the prevalence of typhoid fever is 500 per 1,0 population, and the mortality rate is between 0.6% and 5%.^3^ Lampung Province has the highest number of suspected typhoid cases (244,071 cases), and Bandar Kota Lampung contributed 11,387 cases.^4^

Typhoid fever is caused by the bacteria *Salmonella typhi*, which is spread through fecal contamination of water and food, either through typhoid fever patients or carriers who excrete *Salmonella*.^5^ Given the high prevalence of typhoid, research to investigate typhoid fever's sources and modes of transmission is essential, as socio-demographic, behavioral, and environmental conditions are unique and variable.^6,7^ A detailed understanding of local risk factors for typhoid fever is needed to inform non-vaccine control measures.^2^ The study aims to analyze the risk factors for typhoid fever in Bandar Lampung City, Indonesia.

## METHODS

The study applied the case-control method to compare past exposure between participants who had typhoid fever (cases) and participants who did not have typhoid fever (controls). This study was conducted after obtaining approval from the Health Research Ethics Committee of the Tanjung Karang Health Polytechnic (No. 244/KEPK-TJK/III/2023). According to the Helsinki protocol, consent was obtained, and data handling was confidential. There was no risk of harm to participants, and participants had the right to withdraw during the study. All research procedures were explained before the interview. The data is confidential, and only researchers can access it. In the data entry process, the identities of all respondents were replaced with numbers.

The study was conducted from March 2023 to June 2023 in Kemiling Sub-District, Bandar Lampung City, Indonesia. All residents aged 5-55 years who have permanent residence (minimum six months) were included in the study. Residents who were sick, disabled, traveling, or otherwise unwilling were excluded from the study. The age restriction of participants was to control for potential recall bias.

The cases studied were recovered typhoid fever patients aged 5-55 years who were treated from February to May 2023 at the Kemiling Public Health Center, Bandar Lampung, Indonesia. They had recovered from culture-confirmed infection by *Salmonella typhi* or *S. paratyphi* in the blood and had been discharged two weeks before the study. The blood culture was verified from hospital records.

For each case, we randomly selected control subjects in the residential environment with no history of typhoid fever in the past month. To find suitable controls, a standard procedure was used: First, the house on the right side of the patient's house was selected, and if no control subjects were found that met the criteria, we tried the house on the left side, followed by the second house on the right and so on.

The sample size calculated was the following formula:


$$\text{n}=\frac{\text{2}p\;q{\left(Z_{\alpha }+Z_{\beta }\right)}^{2}}{{\left(P_{\text{1}}-P_{0}\right)}^{2}}$$

where, n= sample size; Z α = specified significance (1.96); Z β = specified power (0.84); p_0_ = proportion ofcontrol with exposure (0.116); p_1_ = proportion of case with exposure= p_0_ R/[1 + p_0_ (R-1)]; p = mean proportion exposure in the case and control group= {(p_0_ + p_1_)/2}; q = mean proportion unexposed in the case and control group= (1 - p); R= Odds Ratio to be detected (3.0).

According to Ulfa F and Handayani O, in the variable eating unwashed raw vegetables (p 0 =11.6%; R= 3.0), 8 and assuming 95% CI, 80% power, control to case ratio 1: 1, the total sample size is 206 (103 cases and 103 controls).^8^

All participants were visited at home. Both cases and controls were interviewed using a questionnaire describing the individual's demographics, socioeconomic conditions, personal hygiene, and food hygiene habits. Structured observations of the house took into account access to sources of safe drinking water and healthy latrines. Interviewers were environmental health students trained before going into the field, supervised during the initial phase, and randomly afterward. Enrolled patients provided informed consent during the last days of their hospitalization, and the agreement of controls was obtained before the interview.

Data collected was entered into Statistical Package for Social Science (SPSS) after being checked for completeness, edited, and coded. Code results are given: 1 for cases and 0 for controls. A chi-square test was used to measure the difference in the proportion of exposure between the case and control groups. Variables with p < 0.25 were forwarded to multivariable analysis using logistic regression tests. Adjusted Odds Ratio (AOR) and 95% confidence interval (95%CI) were applied to assess risk factors. All statistical tests were performed at alpha = 5%, and p ≤ 0.05 was considered statistically significant.

## RESULTS

**Table 1:** Participant's characteristics

Characteristic	Case(n=103)	Control (n=103)
**Age (years)**		
Mean	23.7	23.1
Range	6-51	6-52
**Gender**		
Male	41 (39.81)	41 (39.81)
Female	62 (60.19)	62 (60.19)
**Education level**		
Elementary school	9 (8.74)	21 (20.39)
Junior high school	13 (12.62)	8 (7.76)
Senior high school	43 (41.75)	56 (54.37)
College	38 (36.89)	18 (17.48)

During the study period, 103 cases and 103 controls were interviewed and their living environment was observed. The average age of cases was 23.7 (6-51) years and controls 23.1 (6-52) years. There were 41 (39.81%) male and 62 (60.19%) female participants, in both cases and controls. Out of total, 43 (41.75%) cases and 56 (54.37%) controls had senior high school education (Table 1).

The univariate analysis shows that 88 (85.44%) cases did not always wash their hands with soap after defecating or before eating. In contrast, 65 (63.11%) controls always washing hands with soap after defecating or before eating. About 84 (81.55%) cases did not wash raw vegetables before eating or cooking. This study found that 66 (64.08%) cases usually eat mobile food, and cases eating street food were 78 (75.73%). Mobile food is food that is sold by going around. While street food is food that is sold on the roadside permanently (Table 2).

Based on housing factors, the most of case households did not have access to safe drinking water 91 (88.35%) and healthy latrines 70 (67.96%). Meanwhile, 52 (50.49%) of control households had access to safe drinking water, and 68 (66.02%) had access to healthy toilets. . In this study, access to safe drinking water was assessed based on the construction of drinking water sources, the distance from biological pollutant sources (including septic tanks and open sewage channels), and drinking water treatment (including boiling, reverse osmosis, disinfection). Meanwhile, access to healthy latrines was assessed from the construction of latrines that were always used by participants.

Seven variables were continued into multivariable analysis with logistic regression to determine the risk factors for typhoid fever. The multivariate analysis results found that not washing hands with soap after defecating or before eating was 6.08 (95% CI 2.43 - 15.21; P< 0.001) times more likely to have typhoid than washing hands with soap. Meanwhile, those not washing raw vegetables were 4.36 (95% CI 1.62 - 11.73; P= 0.004) more likely to have typhoid than washing.

This study found that eating mobile food was 10.89 (95% CI 4.08 - 29.05; P< 0.001) times more likely to have typhoid. Meanwhile, street food has a 3.28 (95% CI 1.27 - 8.45; P= 0.014) times greater risk of typhoid (Table 2).

The two environmental variables studied are risk factors for typhoid. Households without access to safe drinking water are 6.08 (95% CI 2.11 - 17.52; P= 0.001) times more likely to have typhoid than those with access to safe drinking water. Meanwhile, households without access to healthy latrines were 3.59 (95% CI 1.47 - 8.78; P= 0.005) times more likely to have typhoid than those with access.

**Table 2:** Univariate and multivariate analysis results

Variables	Groups		Unadjusted OR (95% CI)	p-value	Adjusted OR (95% CI)	p-value
	Case (n=103)	Control (n=103)				
**Washing hands**						
No	88 (85.44)	38 (36.89)	10.03 (5.09-19.77)	0.0001	6.08 (2.43-5.21)	0.0001
Yes	15 (14.56)	65 (63.11)	1		1	
**Washing raw vegetables**						
No	84 (81.55)	46 (44.66)	5.48 (2.91-10.30)	0.0001	4.36 (1.62-11.73)	0.004
Yes	19 (18.45)	57 (55.34)	1		1	
**Eating mobile food**						
Yes	66 (64.08)	16 (15.53)	9.67 (4.97-18.92)	0.0001	10.89 (4.08-29.05)	0.0001
No	37 (35.92)	87 (84.47)	1			
**Eating street food**						
Yes	78 (75.73)	35 (33.98)	6.06 (3.30-11.13)	0.0001	3.28 (1.27-8.45)	0.014
No	25 (24.27)	68 (66.02)	1		1	
**Safe drinking water**						
No	91 (88.35)	51 (49.51)	7.73 (3.78-15.80)	0.0001	6.08 (2.11-17.52)	0.001
Yes	12 (11.65)	52 (50.49)	1		1	
**Healthy latrines**						
No	70 (67.96)	35 (33.98)	4.12 (2.30-7.36)	0.0001	3.59 (1.47-8.78)	0.005
Yes	33 (32.04)	68 (66.02)	1		1	

OR = ODDs Ratio, CI = Confidence Interval

## DISCUSSION

In this study, we found that those who did not wash their hands with soap after defecating and before eating were 6.08 times more likely to have typhoid than those who washed their hands (95% CI; 2.43 - 15.21; P< 0.001). Similar to another study conducted in Semarang (Indonesia), no washing hands with soap independently carries a 4.63 times risk of typhoid fever (95% CI 1.62 - 11.73).^5^ A study done in Jakarta, Indonesia revealed the odds ratio to be 1.91 (1.06-3.46).^9^ While in Fiji, a similar study reported that not washing hands with soap has a 1.63 times risk of getting typhoid fever (95% CI; 1.05 - 2.70).^10^ Washing hands with soap is an essential and fundamental factor in preventing the transmission of typhoid fever.^8,11-15^

Transmission of typhoid occurs via the fecal-oral route, so hygienic measures are involved in most of its control and prevention.^16^ *Salmonella typhi* can survive on the fingers for at least ten minutes. Therefore, washing hands that do not comply with standards (including using soap in running water and rubbing between fingers and nails) allows *Salmonella* to remain and cause typhoid fever.^13^

Not washing raw vegetables (raw consumption) was 4.36 times more likely to lead to typhoid than washing (95% CI; 1.62 - 11.73; P= 0.004). Similar to the study in Fiji, not washing row vegetables had 3.48 (2.065.89) times the risk of typhoid.^10^ In an Indian study, it reported that not washing food properly has 1.83 (1.043.20) times more likely to have typhoid.^12^ Meanwhile, in Karachi (Pakistan), it was found that not washing vegetables properly had a 4.93 (1.58-15.43) times greater to typhus.^11^

Pathogenic microbial contamination of agricultural products occurs at several points, starting from the production, harvest, packaging, storage, processing, distribution, and marketing stages.^17^ In Indonesia, most of the population buy food ingredients in traditional markets that are done openly; these lack sanitation, and proper cold storage.^18^ *Salmonella* contamination can also come from watering plants and washing crops using water contaminated with human feces.^19^ According to a study, the dynamics of *Salmonella typhi* contamination in vegetables or fruit occurs because farmers use human waste fertilizer, use contaminated water when cleaning fruit during harvest, even when the fruit is sold outdoors and is contaminated after being infested with flies or handled by carrier sufferers.^11^

Uncooked or peeled foods are essential carriers of human pathogens. Green leafy vegetables are suitable for microorganism contamination because they can attack internal tissues through lesions or open stomata.^20^ Microorganisms can enter plants through air tissue or root gaps, but only microorganisms adapted to low oxygen will grow and infect plants through the root route.^21^ The peel is a barrier and prevents the entry of microorganisms into the inner tissues. So, if the peel is damaged and holes form, pathogenic microbes can penetrate and grow.^17^ Therefore, it is essential to establish postharvest control procedures to avoid microbial contamination, including good sanitation practices, control of food transport elements, and adequate cooling temperatures.^17^

*Salmonella typhi* in food and drinks on mobile food or street food sold around is related to using contaminated water to wash food and cutlery, ice from unboiled water, or contamination from food handlers.^5,11-13,22-24^ Other studies conclude that typhoid is transmitted through food handlers with carrier status,^6,25^ and house flies.^26^

We investigated the risk of typhoid based on the types of food usually consumed outdoors. The result shows that mobile food and street food are the primary sources of *Salmonella typhi.* Eating mobile food was 10.89 times more likely to experience typhoid (95% CI 4.08 - 29.05; P<0.001). Meanwhile, eating street food was 3.28 times more likely to cause typhoid (95% CI 1.27 - 8.45; P= 0.014). Similar to a study in in Kathmandu (Nepal), street food consumption (AOR: 2.85; 95% CI; 1.4 - 6.0; P=0.006) remained as strong risk factors for typhoid infection.^27^ Meanwhile, in Karachi, Pakistan, reported that eating food from street vendors significantly increased the chance of typhoid by 8.8 times (OR = 8.8; 95% CI: 95% 2.1-36.2).^24^ In Fiji, it was reported that consuming street food had a risk of 1.56 (1.04 - 2.34) for typhoid.^10^

Another study in Karachi, Pakistan it was found that eating street food increased the risk of typhoid by 4.11 (1.77 - 9.54).^22^ In India, it was reported that consuming street food has a 2.06 (1.03-4.12 0.06) times greater chance of getting typhoid.^12^ In Indonesia, the risk values of 1.83 (1.02-3.26) and 3.86 (1.30-11.48) respectively were found.^5,9^

In this study, we found that households that did not have access to safe drinking water and healthy latrines were at risk of having typhoid 6.08 times and 3.59 times, respectively. Similar to our findings, another study in Pakistan found that using contaminated water was significantly associated with an increased likelihood of typhoid (OR= 10.3; 95% CI: 3.4-30.4).^24^ In Fiji it was found that the risk of typhoid in the group without access to safe drinking water and healthy latrines was 3.61 (1.44 - 9.06) and 49.47 (9.42 - 259.92), respectively.^10^ In Nepal, it was found that use of a unhealthy latrine increased the risk of typhoid 4.10 (95%CI; 1.4 - 12.4; P=0.013).^27^ In Jakarta it was found that not having access to healthy latrines increased the risk of typhoid 2.20 (1.06-4.55) times greater.^9^ Meanwhile, in Semarang it was stated that not having access to safe drinking water increases the risk of typhoid by 29.18 (2.12 - 400.8) times.^5^

Typhoid transmission occurs via the fecal-oral route.^16^ Unsafe latrine construction causes contamination of the population's drinking water sources. Furthermore, water contaminated with feces is used daily (including drinking, washing food, and eating utensils).^28,29^ Meanwhile, open defecation is related to the vector's role as a spreader of *Salmonella typhi* germs.^10,30^ Besides improving personal hygiene, improving access to sanitation and safe drinking water is the primary option in controlling typhoid fever, including free access to safe drinking water in slum settlements. In addition, drinking water treatment is an action that can be taken to control typhoid. According to an Indian study, household drinking water treatment (including boiling, filtration, and reverse osmosis) can increase protection against typhoid (OR = 0.45; 95% CI: 0.25- 0.80).^12^

In general, the results of this study found that typhoid infection is related to personal hygiene, contaminated food, and housing sanitation. All three are common problems in many low-middle income countries and are associated with poverty, low education, and migration, in addition to antimicrobial resistance.^1,2^

Our study has several limitations. First, recall bias that could affect potential exposure during the incubation period and social desirability bias for sanitation and hygiene questions. Therefore, we controlled for this by restricting the age of participants (5-55 years) and observing sanitation facilities. Second, there is a possibility of differential bias. However, we trained all interviewers and ensured that cases and controls were questioned in the same way. Third, we did not examine food samples from street vendors and drinking water samples for *S. typhi.* Future studies are expected to include food samples, drinking water samples, and hand rinse samples from street vendors.

## CONCLUSIONS

In general, the results of this study found that typhoid infection is related to personal hygiene, contaminated food, and housing sanitation. All three are common problems in many low-middle income countries and are associated with poverty, low education, and migration, in addition to antimicrobial resistance.
